# MSP-Net: An Effective Multi-Scale Feature-Aware Detection Network for the Detection of Tomato Leaf Diseases

**DOI:** 10.3390/plants15050711

**Published:** 2026-02-26

**Authors:** Feng Kang, Lijin Wang, Huicheng Li, Yuting Su, Ruichen Chen, Qingshou Wu, Yaohua Lin

**Affiliations:** 1 College of Computer and Information Sciences, Fujian Agriculture and Forestry University, Fuzhou 350002, China; 2 Fujian Key Laboratory of Big Data Application and Intellectualization for Tea Industry, Wuyi University, Wuyishan 354300, China; 3 Key Laboratory of Smart Agriculture and Forestry, Fujian Province University, Fuzhou 350002, China; 4 Engineering Research Center of Smart Sensing and Agricultural Chip Technology, Fujian Province University, Fuzhou 350002, China

**Keywords:** tomato leaf disease detection, multi-scale perception, feature enhancement, attention mechanism, edge deployment

## Abstract

To advance automatic tomato leaf disease detection in precision agriculture, this study addresses critical challenges in complex field environments, such as variable lesion scales, background interference, and deployment constraints. We propose MSP-Net, a task-driven detection framework with targeted architectural refinements integrating three specific optimizations. First, a Multi-Scale Perception Convolution Module (MSPCM) is introduced to capture diverse disease features across early-to-late infection stages. Second, SimAM-enhanced C3k2 layers are utilized to suppress background noise and focus on fine-grained lesion cues. Third, a Multi-Scale Feature Enhancement Module (MSFEM) bridges the semantic gap between shallow and deep features to improve fusion efficacy. Furthermore, we construct a lightweight variant, L-MSP-Net, using architectural migration and structured pruning for edge efficiency. Experimental results on the real-world Tomato-Village dataset show that MSP-Net achieves 92.0% mAP@0.5, outperforming the YOLOv11s baseline by 2.0%. L-MSP-Net attains 86.1% mAP@0.5, improving by 3.6% over the lightweight YOLOv11n baseline while reducing parameters by 10.5%, and is successfully deployed on the RK3588 edge platform. Additional cross-dataset experiments on PASCAL VOC and MS COCO evaluate the transferability of the proposed architectural refinements to generic object detection tasks.

## 1. Introduction

Tomato is one of the most widely cultivated and economically important vegetable crops worldwide. Its stable yield and high quality play a vital role in ensuring global food security and supporting regional agricultural development [1]. However, under intensive cultivation systems, tomatoes are highly vulnerable to a variety of diseases [2]. Without timely detection and intervention, these diseases can cause more than 30% direct yield loss, resulting in substantial economic damage to growers [3]. Therefore, developing an efficient and accurate tomato disease detection approach is crucial for sustainable tomato production and reducing economic losses. Because the early symptoms of many diseases first appear on the leaves, evaluating plant health through leaf images provides an effective means of monitoring disease progression [4]. Traditional machine-learning-based methods have achieved noteworthy progress in this field. Narla and Suresh [5] constructed a multi-feature SVM classifier by integrating color, texture, morphological, and edge features, achieving an overall accuracy of 95.4% for 10 disease categories on 3800 tomato leaf images, demonstrating the effectiveness of handcrafted features. Acharya et al. [6] evaluated SVM and LS-SVM using color–texture features for three rice disease types, where LS-SVM achieved 91.3% and 98.9% accuracy on two independent datasets, respectively, significantly outperforming traditional SVM. These studies confirm the feasibility of feature engineering; however, in real agricultural environments, handcrafted feature extractors are highly sensitive to illumination variation and background complexity, and they often struggle to generalize to large-scale datasets, limiting their robustness, scalability, and efficiency.

In recent years, deep-learning-based object detection models, particularly the YOLO (You Only Look Once) family, have become mainstream solutions due to their exceptional balance between accuracy and inference speed. These models are widely applied across various aspects of tomato agriculture, from detecting fruit maturity in greenhouse environments [7] to identifying leaf diseases. To improve their performance in tomato leaf disease detection, numerous enhancements have been proposed. For example, Liu et al. [8] proposed a label-balanced cross-entropy (LBCE-β) loss, replaced the YOLOX backbone with MobileNetV3, and integrated the CBAM attention module, resulting in more accurate disease detection. Lee et al. [9] improved YOLOv5m by incorporating a C3NN module and a bidirectional feature pyramid network (BiFPN), enabling effective identification of tomato leaf diseases. However, these models still struggle under complex field conditions. When the background contains regions visually similar to lesions, distinguishing foreground from background becomes difficult, particularly for small early-stage lesions, and robustness remains limited.

To address complex background interference, Tang et al. [10] proposed PLPNet, which suppresses soil background noise via adaptive convolution and a position-enhanced attention mechanism, achieving 94.1% mAP@0.5 on a custom tomato leaf dataset; yet, the model remains sensitive to overexposed illumination and leaf overlap. Liu and Wang [11] embedded a hybrid attention mechanism into YOLOv5 to improve detection of tomato brown spot disease in challenging scenarios, effectively alleviating performance degradation caused by overlapping leaves. However, their model still struggles with precise localization of multi-scale disease regions across different disease progression stages. To specifically address this scale challenge, other works have proposed tailored feature fusion strategies for tomato disease detection, further demonstrating the potential of multi-scale approaches [12]. Jing et al. [13] introduced an automatic leaf image annotation algorithm and enhanced YOLOv5 with a weighted BiFPN and CBAM, improving recognition accuracy by 2.4%, though fine-grained severity grading remained limited and automatic labeling exhibited boundary deviations in complex lesions. Abulizi et al. [14] proposed DM-YOLO, incorporating deformable convolution and an MPDIoU loss into the YOLOv9 backbone with bidirectional feature fusion. On a custom dataset of 7800 tomato images, DM-YOLO achieved 94.7% mAP@0.5 with 11.6% fewer parameters and 76 FPS inference speed, outperforming YOLOv9/v8/v5 in complex conditions. Chen et al. [15] developed a lightweight model using C2f-Ghost modules, SPD-Conv, and CA attention, achieving 96.2% mAP@0.5 on the PlantVillage-Tomato dataset with a model size of only 3.2 MB and 112 FPS inference speed. This drive for efficient deployment is also seen in other crop disease research, such as the use of LightYOLO for apple leaf disease detection [16]. Although these improvements demonstrate strong fine-grained recognition capabilities, real-world deployment validation remains insufficient.

Overall, despite substantial progress in improving accuracy, enhancing robustness against complex backgrounds, and achieving lightweight design, existing research still faces several unresolved challenges in real-world field environments:

- Extreme variations in disease scales. Current detectors still struggle with targets of drastically different sizes, particularly early-stage tiny lesions in cluttered backgrounds.
- Complex background interference. Uncontrolled field conditions—such as varying illumination, leaf occlusion, and overlapping foliage—exert significant negative impact on detection reliability.
- Fine-grained differences in disease symptoms. Many diseases exhibit highly similar visual patterns, and some symptoms resemble those of healthy leaves, imposing stringent requirements on feature discrimination.
- Lack of practical edge deployment validation. Many high-accuracy models are too computationally heavy for real-world deployment on resource-constrained devices.

To systematically address the aforementioned challenges, this study proposes MSP-Net, a structured architectural refinement of a multi-scale feature-aware detection network based on the high-performance YOLOv11s model. The network focuses on the task-driven structural enhancements’ design and incorporates a series of targeted structural optimizations to enhance detection robustness and precision in real-world scenarios. Furthermore, to promote real-world applicability, we construct a lightweight version, L-MSP-Net, through architectural migration and structured pruning.

The primary contributions of this study are summarized as follows:

- We design a Multi-Scale Perception Convolution Module (MSPCM) that enhances the model’s sensitivity to lesions of varying sizes by leveraging multi-branch convolutional pathways, significantly improving detection across different lesion scales.
- We integrate a parameter-free attention-enhanced module (SimAM_C3k2) that guides the model to focus on key lesion regions while suppressing complex background noise, improving discrimination of subtle fine-grained symptom differences without increasing model size.
- We introduce a Multi-Scale Feature Enhancement Module (MSFEM) that pre-enhances feature streams between the backbone and neck, bridging the semantic gap between shallow and deep features. This effectively mitigates fine-grained inconsistencies between different disease types and between diseased and healthy leaves, thereby improving overall detection performance.
- We develop and validate a high-accuracy model (MSP-Net) and a lightweight variant (L-MSP-Net), and further demonstrate the feasibility of deploying L-MSP-Net on real edge devices, confirming its practical application potential.

## 2. Discussion

While the experimental results demonstrate consistent performance improvements, it is important to examine the proposed architectural refinements beyond numerical metrics. This section provides a structured discussion of MSPCM, SimAM_Bottleneck, and MSFEM from the perspectives of structural motivation, accuracy–complexity trade-offs, and practical deployment implications. By situating these modules within the broader context of tomato leaf disease detection research, we aim to clarify their contributions, applicability, and limitations under real-world agricultural conditions.

### 2.1. Analysis of MSPCM

The primary motivation behind the design of MSPCM is to address the pronounced multi-scale characteristics of tomato leaf disease patterns. To verify the rationality and near-optimality of the chosen combination of 1 × 1, 3 × 3, and 5 × 5 convolution kernels, we conduct a dedicated ablation study in which the standard convolutions in the baseline model are replaced with different MSPCM variants using various kernel-size combinations. The experimental results are summarized in Table 1.

From Table 1, the impact of different receptive-field configurations on detection performance can be clearly observed.

First, the results highlight the necessity of large receptive fields. In Experiment B, which uses only small and medium receptive fields (1 × 1 and 3 × 3), the performance degrades across all metrics compared with the baseline that uses only 3 × 3 convolutions. However, once the 5 × 5 kernel is introduced (Experiments A and C), both mAP@0.5 and mAP@0.5:0.95 are improved, with particularly notable gains (up to +1.3%) in mAP@0.5:0.95. This indicates that a large receptive field capable of capturing global information from extensive lesion regions is crucial for this task; relying solely on small and medium receptive fields is insufficient to model large-scale disease patterns.

Second, the results also verify the superiority of multi-scale feature fusion. Both dual-branch configurations (Experiments A and C) outperform the baseline, whereas the final MSPCM configuration—combining 1 × 1, 3 × 3, and 5 × 5 kernels—achieves the best overall performance among all variants. Specifically, MSPCM attains mAP@0.5 and mAP@0.5:0.95 scores of 90.6% and 66.5%, representing improvements of 0.6 and 1.9 percentage points over the baseline, respectively. Although its F1-score is not the absolute highest, it is only 0.1 lower than the best value, indicating no meaningful degradation in classification quality.

Additionally, from a computational perspective, the large-kernel branch employs depthwise separable convolution, which limits parameter growth while expanding the receptive field. Although MSPCM introduces a moderate increase in parameters compared with the baseline (an increase of 0.452 M), the improvement is achieved without substantial model expansion. This reflects a practical accuracy–complexity trade-off rather than performance gains purely attributable to increased model capacity.

These experiments collectively confirm the rationality of adopting the 1 × 1, 3 × 3, and 5 × 5 kernel combination in MSPCM. By fusing features from three complementary receptive-field scales within a single module, MSPCM enables the network to adaptively perceive lesions of different sizes at the same feature level, thereby making more accurate decisions. This validates both the design choice and the effectiveness of MSPCM for multi-scale disease representation.

### 2.2. Analysis of SimAM_Bottleneck

To evaluate the effectiveness of the proposed SimAM_Bottleneck module and the rationality of its integration strategy, we design two sets of dedicated experiments. The first set focuses on a horizontal comparison between SimAM and several mainstream attention mechanisms. The second set conducts a vertical analysis of the optimal insertion locations of SimAM_Bottleneck within the network.

We select several popular attention modules—including SENet, CBAM, Coordinate Attention (CA), SKNet and SCSA—and embed them into the YOLOv11s baseline using the same integration strategy as SimAM, ensuring a fair comparison. The results are reported in Table 2.

It can be observed that SimAM delivers the best overall performance among all attention mechanisms considered. In particular, SimAM improves mAP@0.5 from 90.0% to 90.4%, representing the largest gain among the compared modules. At the same time, it introduces no additional parameters, as indicated by the unchanged parameter count (9.415 M) relative to the baseline.

These results suggest that SimAM offers the highest performance-to-cost ratio in this tomato leaf disease detection task, thereby justifying its rationality as the attention mechanism of choice in our improvement framework.

It is worth noting that the performance improvement is achieved without increasing the parameter count, indicating that the gain primarily stems from feature reweighting rather than increased model capacity. This further distinguishes structural contribution from computational expansion.

After identifying SimAM as the optimal attention mechanism, we further investigated the best strategy for integrating it into the YOLOv11s network. We tested various embedding schemes: these included selective replacement based on the specific parameters of the C3k2 module (k = True or k = False), as well as replacement based on the module’s location within the network (Backbone only, Neck only). Finally, these schemes were compared with the strategy of globally replacing all C3k2 modules (ALL). The experimental results are shown in Table 3.

As shown in Table 3, although replacing only the C3k = True modules or only the C3k = False modules both resulted in performance improvement, with the latter achieving an mAP@0.5 of 90.3%, when categorized by network position, replacement only in the Neck also boosted mAP@0.5 to 90.2%, whereas replacement in the Backbone showed poor results. Furthermore, the performance gains from these localized or selective optimization strategies were all inferior to the global replacement strategy (ALL), which achieved the highest mAP@0.5 value of 90.4%. This result suggests that a global, consistent optimization strategy leads to clearer and more efficient feature representation at every level of the model, ultimately yielding maximized performance benefits, thereby validating the rationality of our final model design.

### 2.3. Analysis of MSFEM

MSFEM is designed as a refined variant of the classical Receptive Field Block (RFB). To rigorously validate the rationality and advancement of MSFEM, we perform a fair comparison against the original RFB and its asymmetric version RFB_a proposed in the RFB paper. All three modules are embedded at the same location in the YOLOv11s baseline, and evaluated under identical settings. The results are summarized in Table 4.

As shown in Table 4, all three variants improve detection performance compared with the baseline model. RFB_a achieves the highest mAP@0.5:0.95 (68.0%), indicating strong localization capability under stricter IoU thresholds. However, it also introduces the largest number of parameters (11.671 M), reflecting a higher computational cost.

MSFEM attains an mAP@0.5 of 91.4%, which is the highest among the compared variants under this metric, while maintaining a lower parameter count (11.138 M) than both RFB and RFB_a. Although its mAP@0.5:0.95 is slightly lower than that of RFB_a, MSFEM achieves a competitive F1-score (88.4%) and demonstrates balanced improvements across precision and recall.

From a computational perspective, MSFEM increases the parameter count compared with the baseline model, but its scale remains comparable to that of RFB-based structures. The comparison suggests that the observed performance differences are not solely attributable to parameter growth, but are associated with the specific branch configuration and feature enhancement strategy adopted in MSFEM.

Overall, the results indicate that MSFEM provides consistent performance improvements under a similar model scale, reflecting a practical accuracy–complexity trade-off rather than a simple expansion of network capacity.

### 2.4. Verification of the Scalability of the Modules

To verify the portability and scalability of the three proposed modules—MSPCM, SimAM_Bottleneck, and MSFEM—we migrated them separately to two other models, YOLOv10 and YOLOv12, which share a similar architecture and module composition with YOLOv11. We observed the performance change by integrating each module individually into these two models. The experimental results are shown in Table 5.

The experimental results demonstrate that the MSPCM and MSFEM modules still achieve significant performance gains even when integrated into YOLOv10s. Specifically, MSFEM exhibited a notable improvement, boosting the mAP@0.5 by 0.7% and substantially increasing the mAP@0.5:0.95 by 2.6%. The SimAM_Bottleneck module showed relatively stable performance on this model; although it did not improve the F1-Score or mAP@0.5, it still provided an increase of 0.3% in mAP@0.5:0.95.

For the YOLOv12s model, the integration of each module generally resulted in clear improvements across the evaluation metrics compared to the original YOLOv12s architecture. Specifically, in terms of mAP@0.5, MSPCM, SimAM_Bottleneck, and MSFEM achieved improvements of 2%, 2.9%, and 0.6%, respectively. Although the mAP@0.5:0.95 for MSFEM saw a slight decrease of 0.3%, its F1-Score was enhanced by 1.7%.

The collected experimental evidence suggests that the proposed modules maintain consistent performance when integrated into similar detection frameworks, reflecting their practical portability and reliability within related architectural settings.

### 2.5. Comparison with Recent Tomato Leaf Disease Detection Studies

Recent research on tomato leaf disease analysis can be broadly divided into classification-based methods and detection-oriented approaches, with an increasing trend toward lightweight and deployment-aware designs.

Early studies predominantly relied on image-level classification models trained on curated datasets such as PlantVillage, where leaves are typically isolated under controlled backgrounds. Lightweight classification-oriented architectures have also been proposed for agricultural disease diagnosis. For instance, AgrifusionNet introduces a compact multisource fusion network to improve classification efficiency under relatively controlled imaging conditions [17]. While such approaches often report high recognition accuracy, they do not perform lesion localization and therefore do not directly address multi-instance detection, overlapping leaves, or background interference commonly encountered in open-field environments. In contrast, the present work focuses on object detection under realistic field conditions, where multiple disease instances may coexist within a single image and occlusion significantly increases task complexity.

More recent studies have shifted toward YOLO-based object detection frameworks to enhance localization performance in agricultural settings. For example, DM-YOLO integrates dynamic sampling and improved IoU loss strategies into YOLOv9 to improve small lesion detection and boundary localization, reporting an mAP of 86.4% on tomato leaf disease datasets [14]. Similarly, WTAD-YOLO enhances YOLO11 with multi-scale feature refinement and lightweight convolutional modules, achieving mAP@0.5 of 91.7% with approximately 2.32 M parameters [18]. In a related direction, YOLOv11-RCDWD incorporates channel attention and feature refinement mechanisms into YOLOv11 for maize leaf disease detection, emphasizing improved robustness under complex field backgrounds [19]. These studies demonstrate that architectural refinements within the YOLO family can yield measurable performance gains on plant disease detection tasks.

Compared with these approaches, the present work differs in two aspects. First, unlike prior studies that primarily enhance individual components—such as sampling strategies, loss functions, or backbone attention blocks—the proposed framework emphasizes coordinated structural refinement across different feature processing stages. Specifically, MSPCM operates at the feature extraction stage to strengthen multi-scale representation, SimAM_Bottleneck refines channel responses within transformation bottlenecks, and MSFEM conditions feature streams prior to multi-scale fusion. This cross-stage integration is intended to improve representation consistency under complex field conditions rather than optimizing a single module in isolation. In this sense, MSP-Net integrates multi-scale perception (MSPCM), parameter-free attention embedding (SimAM_Bottleneck), and pre-fusion feature enhancement (MSFEM) within a unified structural refinement framework, while explicitly analyzing how each module contributes to the accuracy–complexity trade-off.

Second, beyond reporting algorithm-level improvements, this study develops a dual-model strategy consisting of a high-accuracy MSP-Net and a deployment-oriented lightweight variant, L-MSP-Net. The lightweight model achieves 86.1% mAP@0.5 with only 2.31 M parameters and demonstrates real-time inference performance of approximately 28.71 FPS on an RK3588 edge platform. Unlike evaluations conducted solely on desktop GPUs [20], this hardware-level validation provides practical evidence of feasibility under embedded computing constraints. While several recent works report inference speed on high-performance GPUs, comprehensive validation on resource-constrained edge devices remains comparatively less explored in tomato leaf disease detection studies.

Overall, while recent works primarily pursue incremental accuracy improvements within specific architectural components, the proposed approach positions itself as a task-driven structural refinement that jointly considers detection performance, computational efficiency, and real-world deployment constraints under complex agricultural conditions.

### 2.6. Limitations and Future Work

Although the proposed MSP-Net and L-MSP-Net demonstrate improved detection reliability under complex field conditions, several limitations should be acknowledged.

First, the evaluation is conducted on the publicly released Tomato-Village dataset, which reflects realistic open-field environments but originates from specific geographic regions. The dataset does not provide plant-level or plot-level identifiers, and therefore the train–validation split follows the official image-level protocol to ensure reproducibility. While this setting aligns with the benchmark configuration, potential scene-level correlations between subsets cannot be entirely excluded. Future studies could incorporate grouped splitting strategies, cross-field validation, or cross-season evaluation protocols—when metadata are available—to more rigorously assess generalization across varying agronomic conditions.

Second, certain extreme scenarios remain challenging. While the visualization results indicate that MSP-Net significantly reduces missed detections and false positives compared with the baseline, severe occlusion, highly dense leaf overlap, and very subtle early-stage symptoms may still produce ambiguous lesion boundaries. These cases represent boundary conditions of RGB-based visual detection rather than failure of the proposed modules. Future research may improve robustness through larger cross-region datasets, more fine-grained stage annotations, or enhanced contextual modeling strategies.

Third, the current framework relies solely on RGB imagery. While this design supports low-cost deployment and practical feasibility, visually similar symptoms—such as nutrient deficiencies resembling early fungal infection—may benefit from complementary information. Integrating multimodal inputs, including hyperspectral data, temporal progression sequences, or environmental sensing signals, represents a promising direction for improving discrimination capability in future research.

Fourth, although the lightweight L-MSP-Net achieves real-time performance on an RK3588 edge platform, long-term power consumption, thermal behavior, and operational stability under continuous agricultural deployment were not quantitatively evaluated. Edge devices in field environments are exposed to temperature variation, dust, and unstable power supply. Extended hardware-level benchmarking and real-world field trials would therefore be necessary to validate long-term reliability.

Finally, it should be emphasized that the proposed modules represent task-driven architectural refinements tailored to tomato leaf disease detection rather than fundamentally new learning mechanisms. Future work may explore adaptive feature selection strategies, dynamic inference schemes, or self-supervised pretraining techniques to further enhance cross-crop and cross-dataset robustness.

## 3. Materials and Method

### 3.1. Dataset

The experiments in this study are conducted on the publicly released Tomato-Village object detection dataset (variant-(c)), which was established to reflect real agricultural field conditions. The data were collected from open-field tomato cultivation areas in Rajasthan, India, specifically in the Jodhpur and Jaipur regions. In contrast to laboratory-curated datasets characterized by uniform backgrounds, this dataset contains substantial variability in illumination, foliage density, and background complexity, as well as frequent occlusion and overlapping disease manifestations.

The detection subset originally consists of 1796 annotated field images containing 21,129 labeled instances. According to the dataset documentation, official data augmentation procedures were applied using the Albumentations library, resulting in an expanded set of 14,368 images with 161,223 annotated objects. In this work, we directly utilize the released augmented dataset without introducing additional cross-subset transformations.

Consistent with the benchmark configuration provided by the dataset authors, the data are partitioned into training and validation subsets using an 80%/20% random split, corresponding to 11,493 training images and 2875 validation images. No reorganization or regrouping of samples was performed in order to preserve experimental reproducibility.

The annotated detection categories comprise seven disease-related classes: Early blight, Late blight, Leaf miner, Magnesium deficiency, Nitrogen deficiency, Potassium deficiency, and Spotted wilt virus. Although healthy leaves appear in many images, they are not labeled as independent detection targets.

Many images contain multiple disease instances within a single frame, and overlapping lesions are annotated independently in accordance with standard object detection protocols. Due to natural variation in field occurrence frequency, the dataset exhibits moderate class imbalance across categories. In this study, no explicit class re-weighting or resampling strategies were applied; the model was trained under the default YOLO optimization and batch sampling configuration to maintain consistency with the benchmark setup.

It is important to clarify that the dataset release does not include plant-level or field-plot identifiers. Consequently, splitting strategies based on plant instances or temporal grouping cannot be implemented under the current dataset structure. While image-level partitioning follows the official protocol, potential scene-level correlations between subsets cannot be completely excluded.

**Figure 1:**
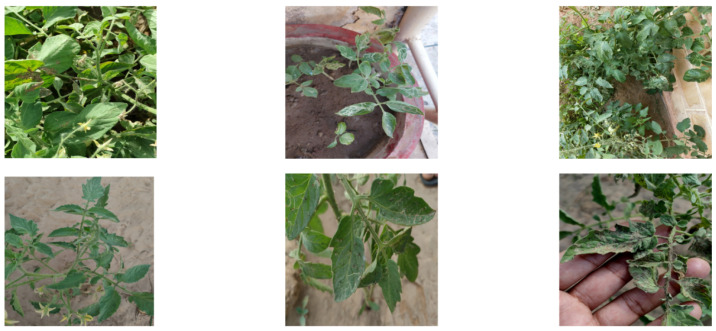
Illustration of the complexity and diversity of the Tomato-Village dataset.

Representative samples of the Tomato-Village dataset are shown in Figure 1, which intuitively illustrate its complexity and diversity in terms of background, illumination, and disease manifestations.

### 3.2. YOLOv11

The YOLO (You Only Look Once) family of algorithms is one of the most influential single-stage object detection frameworks in computer vision, widely adopted due to its superior trade-off between detection speed and accuracy. As one of the latest models in the Ultralytics YOLO series, YOLOv11 [21] inherits and further develops the strengths of its predecessors. Through in-depth optimization of network architecture and training strategies alike, YOLOv11 delivers outstanding overall performance and computational efficiency.

In this study, we select YOLOv11 as the baseline model for further improvement. This choice is primarily motivated by our preliminary comparative experiments on a tomato leaf disease dataset, where YOLOv11 outperformed other models of similar scale, providing a robust foundation for subsequent architectural optimization.

While maintaining the high efficiency characteristic of the YOLO family, YOLOv11 introduces several key architectural refinements, including: (1) C3k2 modules in the backbone and neck. As the core feature extraction component in both the backbone and neck, the C3k2 module is designed to enable in both the backbone and neck networks, aiming to achieve more effective feature recombination and representation compared with previous versions. (2) C2PSA module after the SPPF block. Following the SPPF module, YOLOv11 strategically integrates a C2PSA module. By leveraging a multi-head self-attention mechanism, this module recalibrates and refines aggregated multi-scale features, allowing the network to adaptively emphasize informative channels and spatial regions in a data-driven way, thereby strengthening the capture of key features.

**Figure 2:**
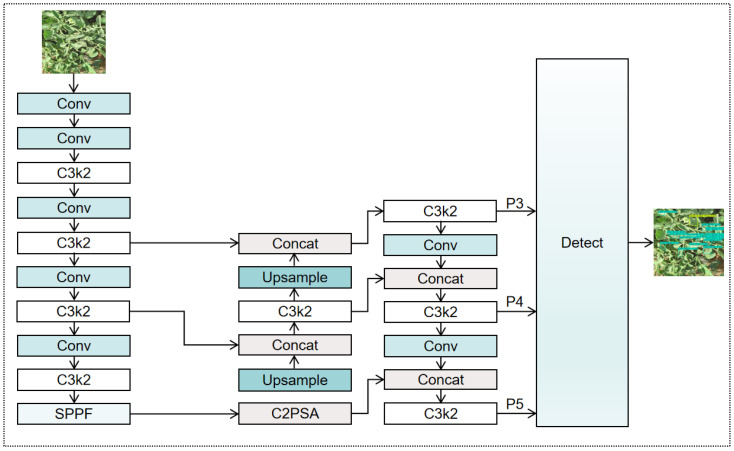
Architecture of YOLOv11.

Despite its strong performance in general object detection tasks, YOLOv11 still exhibits certain limitations when applied to specialized scenarios such as tomato leaf disease detection, where objects exhibit large scale variations, fine-grained symptom differences, and complex backgrounds. To address these issues, we propose a series of targeted improvements based on YOLOv11, as detailed in the following sections. The baseline architecture of YOLOv11 is illustrated in Figure 2.

### 3.3. Multi-Scale Perceptual Convolutional Module

The visual characteristics of tomato leaf diseases exhibit pronounced multi-scale properties. In the early stages of infection, diseases typically manifest as tiny spots (small targets), which may gradually expand into large, irregular lesions (large targets) as the disease progresses. In addition, different types of diseases have distinct typical lesion sizes and shapes. Conventional deep neural networks usually rely on stacked convolution kernels of a single size, whose fixed receptive fields make it difficult to efficiently capture disease patterns across such a wide range of scales. As a result, fine details of small lesions may be lost, while global context information of large lesions may be insufficiently modeled.

To address this issue, we design a task-oriented Multi-Scale Perceptual Convolution Module (MSPCM), which aims to effectively aggregate multi-scale contextual information within a single block and thereby enhance the network’s ability to extract features from lesions of different sizes.

**Figure 3:**
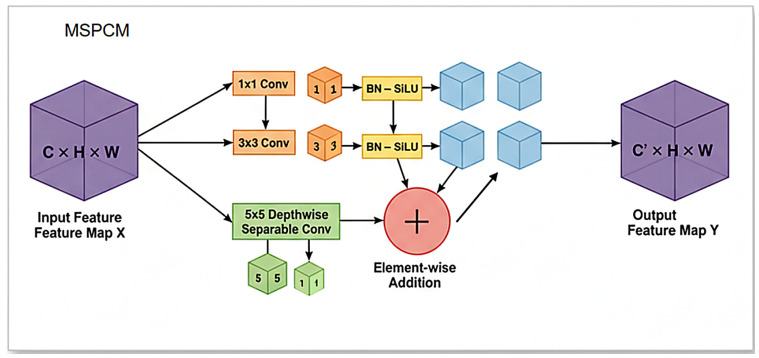
Structure of the MSPCM.

The design of MSPCM is inspired by the classical Inception architecture [22], whose core idea is to increase the network width by employing multiple convolution kernels of different sizes in parallel, so that features at different scales can be captured at the same level. As illustrated in Figure 3, the MSPCM adopts a parallel multi-branch structure. To expand the receptive field effectively while avoiding excessive parameters and computational cost, we introduce a lightweight design strategy: for convolution kernels larger than 3 × 3 (i.e., 5 × 5), we replace standard convolutions with depthwise separable convolutions. This not only reduces the computational burden effectively but also maintains, or even improves, the feature representation capability, achieving a better trade-off between accuracy and model complexity.

The detailed workflow of MSPCM is as follows. Given an input feature map X, the module processes it through three parallel branches: Branch 1 applies a 1 × 1 convolution to capture fine-grained local details, while simultaneously integrating and compressing channel information. Branch 2 employs a standard 3 × 3 convolution to extract medium-scale lesion features. Branch 3 uses a 5 × 5 depthwise separable convolution to obtain a larger receptive field, thereby capturing broader contextual information from large lesions or extensive diseased regions.

Each branch is followed by a Batch Normalization (BN) layer and a SiLU activation function to accelerate convergence and enhance the non-linear representation capability. Finally, the output feature maps of the three branches are fused by element-wise addition to produce the final output feature map Y.

Through this design, MSPCM can adaptively integrate information from multiple receptive fields, substantially improving the representation capability of the network for complex and highly variable disease patterns, and ultimately enhancing overall detection performance.

### 3.4. Simple Parameter-Free Attention Module

Tomato leaf disease detection in complex natural environments poses significant challenges. On the one hand, varying illumination conditions lead to inconsistent visual appearances of lesions. On the other hand, field backgrounds—such as soil, weeds, and overlapping leaves—often resemble lesion textures, resulting in frequent false positives and missed detections. To enhance the model’s feature discrimination capability and robustness under such conditions, we introduce SimAM (A Simple, Parameter-Free Attention Module) [23], a lightweight yet effective attention mechanism.

The key advantage of SimAM lies in its ability to infer a three-dimensional attention weight for every neuron in the feature map without introducing any additional learnable parameters. Inspired by spatial inhibition theory in neuroscience—where informative neurons exhibit firing patterns distinct from their surroundings—SimAM evaluates each neuron’s importance through an energy function. For a given neuron *t* in a feature map $X\in R^{C\times H\times W}$, the energy function $e_{t}^{*}$ is defined as:

(1) $$e_{t}^{*}=\frac{4\left({\hat{\sigma }}_{t}^{2}+\lambda \right)}{{\left(t-{\hat{\mu }}_{t}\right)}^{2}+2{\hat{\sigma }}_{t}^{2}+2\lambda }$$

where *t* is the value of the target neuron, ${\hat{\mu }}_{t}$ and ${\hat{\sigma }}_{t}^{2}$ denote the mean and variance of all other neurons within the same channel (representing global contextual statistics), λ is a regularization hyperparameter used to prevent numerical instability.

According to spatial inhibition theory, a lower energy value $e_{t}^{*}$ indicates a larger difference between the target neuron and its surrounding neurons, implying higher importance. Therefore, the inverse of $e_{t}^{*}$ is used as a measure of neuron significance.

SimAM computes an attention map by normalizing these values through a Sigmoid function and applies it to the input feature map via element-wise multiplication:

(2) $$\tilde{X}=\mathrm{sigmoid}\;\left(\frac{1}{E}\right)⊙X$$

where *E* denotes the set of energy values of all neurons, ⊙ represents element-wise multiplication, $\tilde{X}$ is the enhanced feature map after attention weighting.

Inspired by another model we proposed—PC-YOLO [24]—to integrate SimAM effectively into the YOLOv11 architecture, we first construct a SimAM_Bottleneck module and then fuse it with the existing C3k2 structure to develop a new module named SimAM_C3k2. The core idea is to leverage SimAM to compensate for the potential loss of fine-grained lesion information during the channel compression–expansion process of the bottleneck, guiding the network to focus more accurately on crucial lesion regions and suppress irrelevant background noise. This results in improved detection robustness and precision under complex field conditions.

Compared with existing C3 or C3k2 variants that attach attention modules after feature aggregation, our design adopts a structural embedding of SimAM within the bottleneck block. This allows attention weighting to operate during the channel compression and expansion process, helping retain fine-grained lesion features.

**Figure 4:**
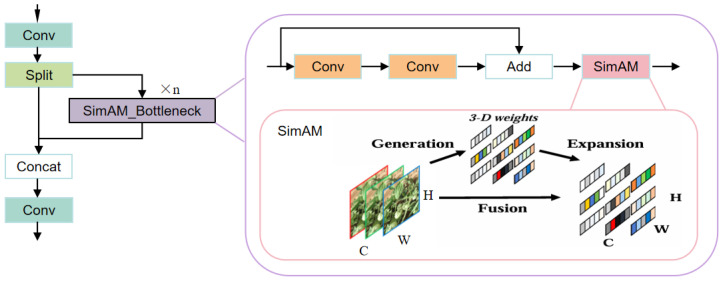
Structure of the SimAM_C3k2 module, where C3k2 = False.

The architecture of the enhanced SimAM_C3k2 module is illustrated in Figure 4.

### 3.5. Multi-Scale Feature Enhancement Module

The multi-scale feature fusion mechanism in YOLO models combines a Feature Pyramid Network (FPN) with a Path Aggregation Network (PAN) to facilitate interaction across different feature hierarchy levels. However, this design inherently suffers from information imbalance: shallow feature maps retain high-resolution spatial details but lack rich semantic information, whereas deep feature maps contain strong semantic cues but lose fine-grained details due to repeated downsampling. To alleviate this contradiction and ensure that all feature streams entering the FPN and PAN contain discriminative information, we propose the Multi-Scale Feature Enhancement Module (MSFEM).

**Figure 5:**
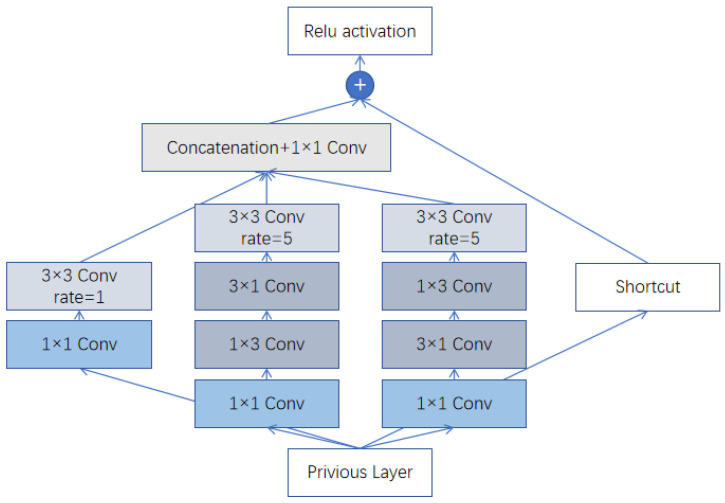
Structure of the MSFEM.

MSFEM is a task-adapted refinement of the Receptive Field Block (RFB) [25], designed specifically for tomato leaf disease detection under complex field conditions. By incorporating parallel dilated convolutions with different dilation rates and asymmetric convolutions, it emulates the receptive field mechanisms of the human visual cortex and aims to enrich both the diversity and representational capacity of features. The architecture of MSFEM is illustrated in Figure 5.

Given an input feature map $X\in R^{C\times H\times W}$, MSFEM processes it through three parallel branches for feature extraction and enhancement. To balance model performance and computational efficiency, each branch begins with a 1 × 1 convolution for channel reduction. The branches are structured as follows:

Branch 1: A standard 3 × 3 convolution to capture fundamental local spatial features. Branch 2: A sequence of 1 × 3 and 3 × 1 asymmetric convolutions to acquire long-range spatial dependencies, followed by a 3 × 3 dilated convolution with a dilation rate of 5. This significantly expands the receptive field with minimal additional parameters, enabling the branch to capture richer contextual information. Branch 3: Similar to Branch 2, but with the asymmetric convolutions reversed (3 × 1 followed by 1 × 3). This complementary design ensures directional diversity and improves the module’s ability to adapt to irregularly shaped lesions.

After feature extraction, the outputs of all three branches are concatenated along the channel dimension:

(3) $$F\;=\;[X_{0},\;X_{1},\;X_{2}]$$

and then fused through a 1 × 1 convolution. To preserve the original feature information and accelerate convergence, we introduce a shortcut connection (residual path). The final output of MSFEM is computed as:

(4) $$Y=\mathrm{ReLU}\;\left(\alpha \cdot {\mathrm{Conv}}_{1\times 1}\left(\left[X_{0},\;X_{1},\;X_{2}\right]\right)+\mathrm{Shortcut}\left(F\right)\right)$$

where α is a scaling factor (set to 0.1 in this study), Shortcut(F) denotes the residual mapping, ReLU is the activation function.

While classical RFB modules focus on enlarging receptive fields for general object detection, MSFEM is configured and positioned to pre-condition feature streams before multi-scale fusion in the YOLO architecture. The complementary asymmetric branch design further adapts the module to irregular lesion patterns.

Subsequently, we insert MSFEM modules between the Backbone and Neck networks to pre-enhance the feature streams before multi-scale aggregation. Specifically: For shallow features, MSFEM supplements missing semantic cues while maintaining high-resolution spatial details. For deep features, MSFEM helps restore fine-grained details lost during aggressive downsampling, particularly improving the representation of small lesions.

This design significantly enhances the quality of features entering the Neck for multi-scale fusion, thereby improving detection accuracy, robustness, and generalization capability.

**Figure 6:**
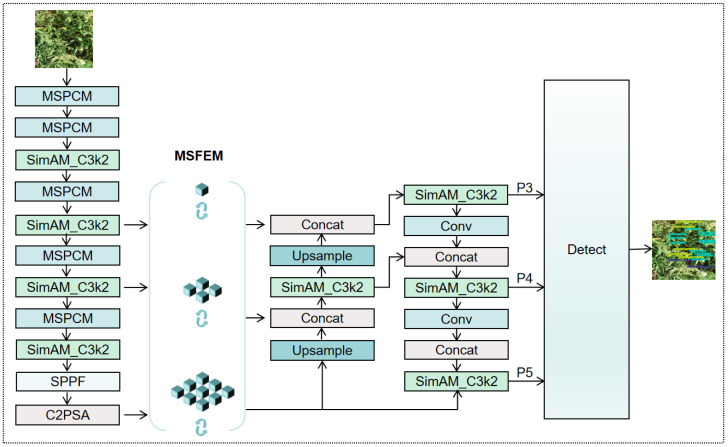
Overall architecture of MSP-Net.

By combining the proposed MSPCM, SimAM_C3k2, and MSFEM modules, we build the final architecture of MSP-Net, as illustrated in Figure 6.

### 3.6. A Lighter Detection Model Based on Pruning

In the YOLO family of object detection models, the s-scale (small) variant is often used as a baseline for algorithmic verification, since its moderate depth and complexity make it suitable for evaluating the generalization ability and performance upper bound of newly designed modules. However, for practical deployment on resource-constrained edge devices (e.g., unmanned aerial vehicles, mobile robots), the n-scale (nano) models are usually preferred due to their extremely lightweight architectures.

To achieve both high performance and efficient deployment, we adopt a “validate first, compress later” strategy. As described earlier, we integrate the proposed MSPCM, SimAM_C3k2, and MSFEM modules into the YOLOv11s baseline to construct a high-performance detection model, MSP-Net. After thoroughly validating the effectiveness of MSP-Net, we transfer the same architectural improvements to the more lightweight YOLOv11n backbone, aiming to balance detection accuracy and deployment efficiency.

To further optimize inference speed and reduce model size, we apply structured pruning, specifically channel pruning, to the enhanced n-scale model. This technique reduces the number of feature maps in each convolutional layer by removing entire convolutional kernels [26]. Compared with unstructured pruning, structured pruning produces a more hardware-friendly and deployment-ready compressed model. In this work, the importance of each channel is measured using the gamma (γ) coefficients of the Batch Normalization layers. Channels with small γ values are considered less informative and are pruned as redundant components. After pruning, we perform fine-tuning, retraining the remaining network parameters and convolutional kernels to restore, as much as possible, the performance of the original model. This process effectively preserves key semantic information while significantly reducing the number of parameters and computational complexity (GFLOPs).

Through the above lightweight design, we finally constructed a deployment-oriented model named L-MSP-Net (Lightweight MSP-Net). L-MSP-Net inherits the same architectural ideas as MSP-Net but has a much smaller model size, achieving an excellent accuracy–efficiency trade-off and laying a solid foundation for subsequent real-world deployment on edge devices.

## 4. Experiment

To comprehensively evaluate the effectiveness of the proposed MSP-Net, a series of controlled experiments are conducted on the Tomato-Village object detection benchmark described in the previous section. The evaluation includes overall model comparison, module-level ablation analysis, and performance complexity assessment.

In addition, to examine whether the proposed architectural refinements remain effective beyond the tomato disease domain, we further conduct cross-dataset transfer experiments on two widely used object detection benchmarks, MS COCO and PASCAL VOC 2012. These experiments are intended to assess architectural compatibility rather than to claim agricultural cross-domain generalization.

Finally, the lightweight variant L-MSP-Net is deployed on an RK3588-based embedded development board, and its inference performance is evaluated to verify practical feasibility under resource-constrained edge conditions.

### 4.1. Experimental Environment

All experiments in this study are implemented using the PyTorch deep learning framework v2.9.0 with Python 3.10. Experimental platform hardware environment: The CPU is 14vCPU Intel(R) Xeon(R) Gold 6330, the GPU is NVIDIA GeForce RTX 3090 24G, the memory is 90GB, and the operating system is 64 bit Ubuntu. The specific parameter settings are as follows: the input image size is 640 × 640, batch size is 32, and the SGD optimizer is used with an initial learning rate of 0.001, momentum of 0.937, and weight decay of 0.0005. The IoU threshold for positive sample assignment is set to 0.7.

To ensure experimental stability, the key models (YOLOv11s, YOLOv11n, MSP-Net, and L-MSP-Net) were trained three times under identical configurations. The performance variance across runs was within ±0.2% mAP, indicating stable convergence. Because the initial repetitive tests showed extremely small performance fluctuations, only a single run was conducted for the ablation experiment and the auxiliary experiment.

### 4.2. Model Evaluation Metrics

To comprehensively and objectively evaluate the performance of the proposed models, we adopt Precision, Recall, F1-score, and mean Average Precision (mAP) as evaluation metrics.

Precision measures the proportion of correctly predicted positive samples among all samples predicted as positive, and is defined as:

(5) $$\mathrm{Precision}=\frac{\mathrm{TP}}{\mathrm{TP}+\mathrm{FP}}$$

Recall measures the proportion of correctly detected positive samples among all actual positive samples, and is defined as:

(6) $$\mathrm{Recall}=\frac{\mathrm{TP}}{\mathrm{TP}+\mathrm{FN}}$$

where TP (true positives) denotes the number of samples that are actually positive and correctly predicted as positive, FP (false positives) denotes the number of samples that are actually negative but incorrectly predicted as positive, and FN (false negatives) denotes the number of samples that are actually positive but incorrectly predicted as negative.

The F1-score is the harmonic mean of Precision and Recall, providing a balanced assessment of both metrics:

(7) $$F1=2\cdot \frac{\mathrm{Precision}\cdot \mathrm{Recall}}{\mathrm{Precision}+\mathrm{Recall}}$$

For object detection tasks, Average Precision (AP) and mean Average Precision (mAP) are widely used. AP is computed as the area under the Precision–Recall (PR) curve for a given category:

(8) $$\mathrm{AP}=\int_{0}^{1}P\left(R\right)\;dR$$

where P(R) denotes the precision at recall level *R*. The overall performance across all categories is then quantified by mAP:

(9) $$\mathrm{mAP}=\frac{1}{N}\sum_{i=1}^{N}{\mathrm{AP}}_{i}$$

where *N* is the number of categories (set to 8 in this study), and $AP_{i}$ is the Average Precision for the i-th category. These metrics jointly reflect the model’s classification accuracy and localization capability across multiple disease types.

### 4.3. Comparative Experiments

In this section, to validate the effectiveness of the proposed model, we compare MSP-Net with several state-of-the-art (SOTA) object detectors, including Transformer-based models RT-DETR [27], DEIM-RT-DETR and DEIM-DFINE [28], as well as multiple representative models from the YOLO family [29,30,31,32,33,34]. All models are trained and evaluated under the same experimental settings for a fair comparison.

The final detection performance of each model on the Tomato-Village validation set is summarized in Table 6. The boldfaced entries correspond to the proposed model and its results.

From Table 6, it can be observed that the proposed MSP-Net achieves the highest scores among the compared models under the evaluated metrics. The detailed analysis is as follows:

Among all competing models, YOLOv11s exhibits the strongest baseline performance, with mAP@0.5 and mAP@0.5:0.95 reaching 90.0% and 64.6%, respectively, which are clearly surpass other YOLO variants and SOTA Transformer-based detectors. This confirms the intrinsic architectural merits of YOLOv11s and validates its selection as the baseline model in this study, ensuring that subsequent improvements are evaluated on a strong foundation.

The Transformer-based models DEIM-RT-DETR and DEIM-DFINE achieve high recall rates (89.2% and 87.9%, respectively), but their precision is relatively lower, indicating that they tend to confuse complex background regions with true lesion areas. Notably, YOLOv12s shows an extremely high precision (98.5%) but a very low recall (40.3%), resulting in a much lower mAP@0.5 (69.4%) compared with mainstream models. The unusually low recall of YOLOv12s under our experimental configuration might be attributed to its architectural characteristics that prioritize high-confidence predictions—its attention mechanism and R-ELAN feature aggregation module tend to retain high-confidence detection results and suppress low-confidence proposals, thereby resulting in fewer effective detection proposals on the tested dataset.

Building on the strong YOLOv11s baseline, the proposed MSP-Net achieves a consistent performance improvement across all metrics. Specifically, MSP-Net attains a precision of 92.0%, recall of 87.1%, and F1-score of 89.4%, which are 1.3, 1.8, and 1.5 percentage points higher than those of YOLOv11s, respectively, indicating a more favorable balance between precision and recall. In terms of overall detection performance, MSP-Net improves mAP@0.5 from 90.0% to 92.0% and mAP@0.5:0.95 from 64.6% to 67.8%, yielding the strongest performance among the tested configurations on the Tomato-Village dataset.

In summary, although the proposed architectural enhancements lead to a moderate increase in the number of parameters, the performance gains of MSP-Net are substantial and comprehensive. The experimental results indicate that the refinements contribute positively to tomato leaf disease detection in complex natural environments.

### 4.4. Ablation Experiments

To systematically analyze the effectiveness of each proposed module and their combined synergistic effects, we conduct a series of ablation experiments on the Tomato-Village dataset. Starting from the baseline YOLOv11s model, we gradually add individual modules and their combinations to evaluate their respective contributions to the final MSP-Net. The results are summarized in Table 7.

As shown in Table 7, merging all the proposed modules into the benchmark model can improve the detection performance of the model to varying degrees.The detailed analysis is as follows:

When MSPCM is introduced alone, mAP@0.5 increases by 0.6 percentage points (from 90.0% to 90.6%), and the more stringent mAP@0.5:0.95 improves by 1.9 percentage points (from 64.6% to 66.5%). This indicates that the multi-branch, multi-kernel design of MSPCM effectively helps the network capture multi-scale lesion features across different stages and disease types.

Integrating SimAM_C3k2 guides the model to focus more on key lesion regions while suppressing background interference, leading to a 0.4 percentage point gain in mAP@0.5. However, mAP@0.5:0.95 drops slightly by 0.4 percentage points. This suggests that, while the module enhances feature saliency, it may also perturb certain boundary details: SimAM_C3k2 tends to concentrate attention on the most salient lesion cores, reducing its emphasis on blurry or transitional edge regions where lesions gradually merge into the background. Consequently, performance under high IoU thresholds is mildly affected. Nevertheless, subsequent combination experiments show that this drawback is effectively compensated when SimAM_C3k2 works together with other modules.

Benefiting from asymmetric and dilated convolutions, MSFEM substantially enriches the receptive field and contextual information of features before they flow into the Neck for fusion. It effectively alleviates the lack of semantics in shallow features and the loss of fine details in deep features. As a result, mAP@0.5 and mAP@0.5:0.95 increase by 1.4 and 1.9 percentage points, respectively, representing one of the most significant single-module gains.

Overall, different combinations of MSPCM, SimAM_C3k2, and MSFEM all yield further improvements over the baseline, indicating that the modules are largely complementary. When all three modules are integrated, the resulting MSP-Net achieves the best overall performance, with mAP@0.5 and mAP@0.5:0.95 reaching 92.0% and 67.8%, respectively—improvements of 2.0 and 3.2 percentage points over the baseline YOLOv11s. This confirms the strong synergistic interaction among the modules and shows that they collectively contribute to a qualitative enhancement in detection performance.

To more intuitively validate the effectiveness of MSP-Net in capturing key lesion features, we further conduct visualization analyses from two perspectives: internal attention patterns and final detection outputs.

**Figure 7:**
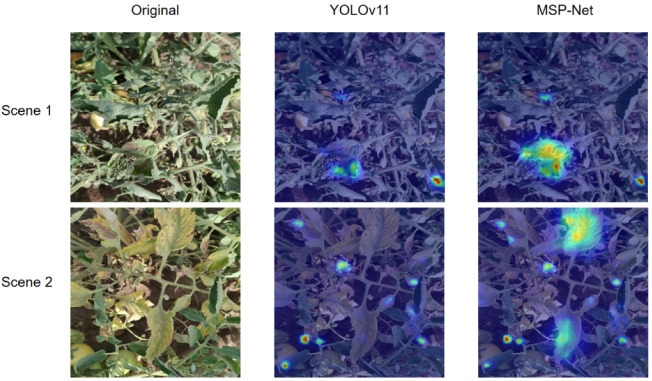
Comparison of attention heatmaps between YOLOv11s and MSP-Net.

First, we generate feature-level heatmaps to visualize the regions the model focuses on during detection. As illustrated in Figure 7, the baseline YOLOv11s tends to exhibit relatively dispersed attention and is easily disturbed by complex backgrounds, whereas MSP-Net can focus more accurately on the true lesion regions, reflecting its superior capability in perceiving discriminative target features.

**Figure 8:**
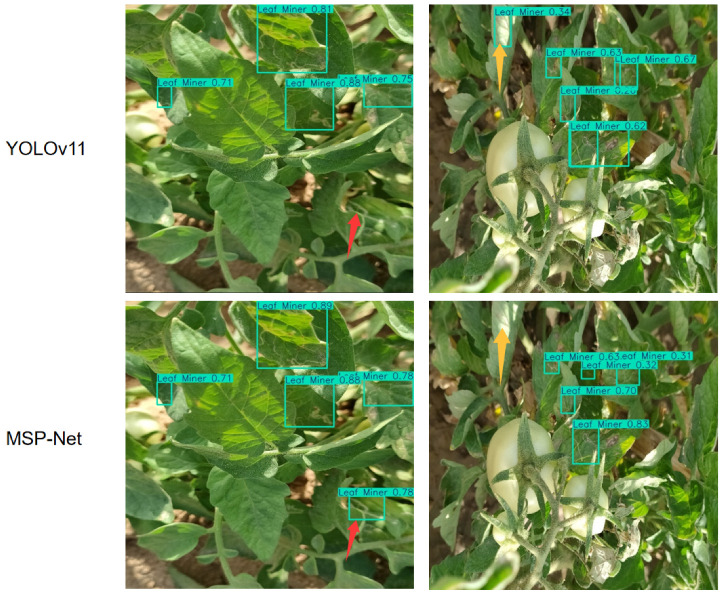
Comparison of final detection results between YOLOv11s and MSP-Net. Red arrows indicate missed detections, and yellow arrows indicate false detections.

This improvement in internal feature perception ability is ultimately directly reflected in the accuracy of the detection results. As shown in Figure 8, we further compare the final detection outputs of the two models under challenging conditions with cluttered backgrounds and visually similar healthy and diseased leaves. The baseline model suffers from missed detections (highlighted by red arrows) and false detections, where healthy leaves are incorrectly identified as diseased regions (highlighted by yellow arrows). In contrast, MSP-Net, empowered by its enhanced fine-grained feature discrimination and focused attention mechanisms, successfully overcomes these interferences and achieves precise lesion localization and recognition.

Taken together, the visualizations in Figure 7 and Figure 8 provide strong evidence that MSP-Net improves detection reliability in complex natural environments, greatly reducing the likelihood of missed and false detections in tomato leaf disease monitoring.

### 4.5. Cross-Dataset Transfer Evaluation of MSP-Net

To further evaluate whether the proposed architectural refinements remain effective beyond the Tomato-Village dataset, we conduct additional experiments on two widely used object detection benchmarks, MS COCO and PASCAL VOC 2012. These datasets differ substantially from tomato leaf disease detection in terms of visual semantics and label space. Therefore, the purpose of this evaluation is to examine architectural transferability. Under identical training settings, we conduct a fair comparison between MSP-Net and the baseline YOLOv11s model. The experimental results are summarized in Table 8.

As shown in Table 8, MSP-Net achieves slightly higher detection metrics than the baseline on both benchmarks. On the COCO dataset, MSP-Net attains mAP@0.5 and mAP@0.5:0.95 scores of 60.1% and 44.1%, representing improvements of 0.7% and 0.8% percentage points over YOLOv11s, respectively. On the PASCAL VOC dataset, MSP-Net yields gains of 0.4% percentage points in both mAP@0.5 and mAP@0.5:0.95 compared with the baseline model.

Although the performance improvements are relatively modest, they are consistent across both datasets. This observation suggests that the proposed multi-scale and feature-enhancement modules are compatible with generic object detection architectures. These findings suggest the potential of MSP-Net for architectural transfer across related detection benchmarks.

### 4.6. Deployment in Edge Devicesn

To apply the proposed method to real-world agricultural scenarios and enable real-time tomato disease detection, we construct a more lightweight model, L-MSP-Net, by combining module transplantation with structured pruning. This process aims to resolve the contradiction that the standard lightweight model (YOLOv11n) lacks sufficient accuracy, whereas the high-performance model (YOLOv11s) is too large for deployment on resource-constrained edge devices. The performance comparison of models at different lightweight stages is listed in Table 9.

As shown in Table 9, our lightweight strategy achieved significant results. Initially, switching from the high-performance YOLOv11s to the lightweight YOLOv11n drastically reduced the parameter count by 72.6%, but the mAP@0.5 also sharply dropped by 7.5 percentage points, indicating a severe loss in model performance. To compensate for this performance gap, we migrated all improvement modules validated on the YOLOv11s model (MSPCM, SimAM_C3k2, MSFEM) to the YOLOv11n model architecture, resulting in MSP-11n. This model successfully raised the mAP@0.5 to 85.6%, recovering 3.1% of the accuracy loss, thus proving the excellent effectiveness of our improved strategy on compact networks as well.

However, the parameter count of MSP-11n (3.134 M) increased compared to the original YOLOv11n. Therefore, we performed structured pruning on it. The progressive process of the pruning operation is shown in Table 10.

As shown in Table 10, when the pruning rate was 40%, the model accuracy reached its highest (mAP@0.5 was 86.4%). However, its parameter count and computational complexity were close to the lightweight baseline model YOLOv11n, failing to provide a offer a meaningful edge in deployment feasibility. Based on an engineering trade-off between accuracy and resource consumption, and considering the limited hardware resources of edge devices, we ultimately selected 50% as the structured pruning rate to obtain the final L-MSP-Net.

Finally, the L-MSP-Net model has a parameter count of only 2.313 M, which is 10.5% smaller than the original YOLOv11n. While reducing computation and storage overhead, its mAP@0.5 remains higher by 3.6%, reaching 86.1%. The pruning process not only effectively eliminated redundant parameters but also promoted more efficient expression by key neurons, fully demonstrating that the synergistic effect of module migration and appropriate pruning holds clear engineering value for edge scenarios.

**Figure 9:**
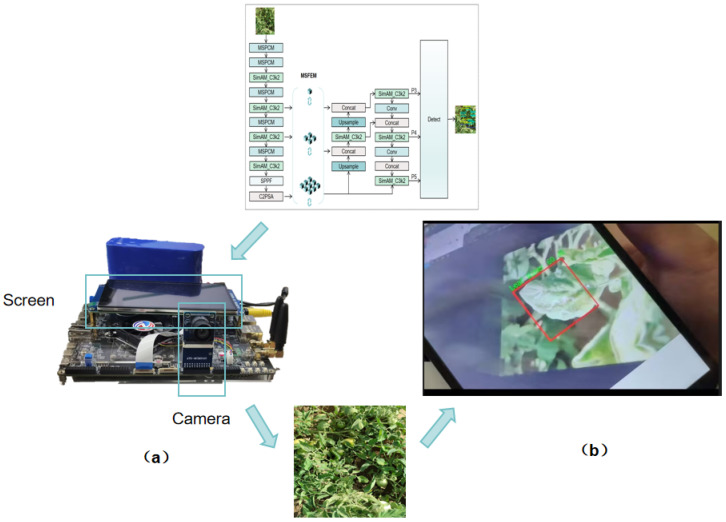
(**a**) Deployment environment of L-MSP-Net on the RK3588 development board; (**b**) real-time detection results on the edge device.

Subsequently, we converted the model to the ONNX format and utilized the RKNN toolkit for quantization and compilation [35]. It was ultimately deployed successfully on the Rockchip RK3588 embedded development board, achieving real-time detection of tomato leaf disease images. This successful deployment validates the practical application value of the lightweight model proposed in this paper. The deployed edge device and detection results are shown in Figure 9.

Lastly, we benchmark the end-to-end runtime performance of L-MSP-Net with batch size = 1 and an input resolution of 640 × 640. After warm-up iterations to exclude initialization overhead, we report the average end-to-end inference latency over repeated runs. L-MSP-Net achieves an average latency of 34.83 ms, corresponding to 28.71 FPS. These results indicate that the proposed lightweight model can meet practical real-time requirements for edge-based tomato leaf disease monitoring.

## 5. Conclusions

This study addresses the practical challenges of tomato leaf disease detection in complex open-field environments, including multi-scale lesion variation, background interference, fine-grained symptom differences, and constraints imposed by resource-limited deployment platforms. Building upon the YOLOv11 framework, we propose MSP-Net as a task-driven architectural refinement that enhances feature perception, attention-guided representation, and pre-fusion feature conditioning within a unified detection pipeline.

The proposed modules—MSPCM, SimAM_C3k2, and MSFEM—are designed as coordinated structural enhancements rather than fundamentally new learning mechanisms. Through systematic integration and analysis, these refinements improve feature consistency across multiple processing stages and demonstrate a balanced relationship between accuracy and computational complexity in tomato leaf disease detection. Furthermore, by transferring the architectural improvements to a lightweight backbone and applying structured pruning, we develop L-MSP-Net to facilitate practical edge deployment under real agricultural constraints.

Beyond algorithmic performance improvements, this work highlights the importance of jointly considering detection reliability, structural efficiency, and deployment feasibility in smart agriculture applications. The results suggest that task-oriented architectural refinement can serve as an effective strategy for improving object detection performance in crop disease monitoring scenarios characterized by occlusion, background clutter, and scale diversity.

Future work will extend evaluation to additional field datasets and protocols, strengthen robustness in highly occluded or visually ambiguous cases, and further benchmark long-term on-device behavior (e.g., efficiency and stability) across heterogeneous edge platforms.

## Figures and Tables

**Table 1:** Effect of different kernel-size combinations on model performance.

Experiment	Kernel Size	P/%	R/%	F1-Score	mAP@0.5/%	mAP@0.5:0.95/%	Params/M
baseline	3×3	90.7	85.3	87.9	90.0	64.6	9.415
A	1×1,5×5	91.0	**85.5**	**88.1**	90.2	65.9	**7.930**
B	1×1,3×3	90.4	83.3	86.7	89.4	64.3	9.632
C	3×3,5×5	**92.0**	83.5	87.5	90.3	65.9	9.651
**MSPCM**	1×1,3×3,5×5	91.1	85.2	88.0	**90.6**	**66.5**	9.867

**Table 2:** Effect of different attention mechanisms on model performance.

Model	P/%	R/%	F1-Score	mAP@0.5/%	mAP@0.5:0.95/%	Params/M
YOLOv11s	90.7	**85.3**	87.9	90.0	64.6	9.415
SENet	90.4	83.7	86.9	89.5	**65.0**	9.430
CBAM	90.9	84.8	87.7	90.2	64.9	9.533
CA	90.4	82.7	86.3	89.3	64.4	9.454
SKNet	90.4	83.4	86.7	89.8	64.8	13.454
SCSA	90.5	82.9	86.5	89.2	64.4	9.432
**SimAM**	**91.4**	85.1	**88.1**	**90.4**	64.2	**9.415**

**Table 3:** Effect of different insertion locations of SimAM on model performance.

Location	P/%	R/%	F1-Score	mAP@0.5/%	mAP@0.5:0.95/%	Params/M
YOLOv11s	90.7	**85.3**	87.9	90.0	64.6	9.415
C3k = Ture	91.3	84.1	87.5	90.1	**65.8**	9.415
C3k = False	91.3	84.3	87.6	90.3	64.5	9.415
Backbone	90.6	84.4	87.3	89.9	64.9	9.415
Neck	91.4	83.7	87.3	90.2	64.9	9.415
**ALL**	**91.4**	85.1	**88.1**	**90.4**	64.2	**9.415**

**Table 4:** Performance comparison between MSFEM and RFB-series modules.

Model	P/%	R/%	F1-Score	mAP@0.5/%	mAP@0.5:0.95/%	Params/M
YOLOv11s	90.7	85.3	87.9	90.0	64.6	9.415
RFB	91.2	85.0	87.9	91.0	66.9	11.331
RFB_a	91.5	85.4	88.3	91.2	68.0	11.671
**MSFEM**	91.1	85.9	88.4	91.4	66.5	11.138

**Table 5:** Verification of the portability of the proposed modules.

Model	MSPCM	SAM_Bot *	MSFEM	P/%	R/%	F1-Score	mAP@0.5/%	mAP@0.5:0.95/%	Params/M
YOLOv10s				89.0	82.5	85.6	88.9	64.3	8.041
	✓			90.5	82.3	86.2	89.4	65.6	8.065
		✓		88.7	82.8	85.6	88.9	64.6	8.041
			✓	**91.0**	**83.1**	**86.8**	**89.6**	**66.9**	9.656
YOLOv12s				98.5	40.3	57.1	69.4	58.6	9.077
	✓			97.9	45.1	61.7	71.4	60.4	9.487
		✓		**99.1**	**45.4**	**62.2**	**72.3**	**60.7**	9.077
			✓	98.1	42.0	58.8	70.0	58.3	9.365

* SAM_Bot is an abbreviation of SimAM_Bottleneck.

**Table 6:** Performance comparison of different models on the Tomato-Village validation set.

Model	P/%	R/%	F1-Score	mAP@0.5/%	mAP@0.5:0.95/%	Params/M
RT-DETR-L	88.3	80.4	84.1	85.8	57.0	32.000
DEIM-RT-DETR	79.4	89.2	84.0	86.3	56.5	20.000
DEIM-DFINE	80.3	87.9	83.9	87.7	58.6	10.000
YOLOv3-tiny	87.3	68.5	76.7	77.7	51.1	9.523
YOLOv5s	90.1	82.5	86.1	88.4	61.7	7.816
YOLOv6s	87.2	74.3	80.2	82.8	58.2	15.977
YOLOv8s	90.2	82.3	86.0	88.5	62.6	9.830
YOLOv9s	89.3	80.6	84.7	88.4	62.9	**6.196**
YOLOv10s	89.0	82.5	85.6	88.9	64.3	8.041
YOLOv11s	90.7	85.3	87.9	90.0	64.6	9.415
YOLOv12s	98.5	40.3	57.1	69.4	58.6	9.077
YOLOv13s	87.1	79.8	83.2	88.7	64.0	9.004
**MSP-Net (Ours)**	**92.0**	**87.1**	**89.4**	**92.0**	**67.8**	11.590

**Table 7:** Ablation study of different modules on the Tomato-Village validation set.

Model	MSPCM	SimAM_C3k2	MSFEM	P/%	R/%	F1-Score	mAP@0.5/%	mAP@0.5:0.95/%	Params/M
11s				90.7	85.3	87.9	90.0	64.6	**9.415**
	✓			91.1	85.2	88.0	90.6	66.5	9.867
		✓		91.4	85.1	88.1	90.4	64.2	9.415
			✓	91.1	85.9	88.4	91.4	66.5	11.138
	✓	✓		90.7	86.0	88.2	90.6	65.8	9.867
	✓		✓	91.9	85.9	88.7	91.5	67.7	11.590
	✓	✓	✓	**92.0**	**87.1**	**89.4**	**92.0**	**67.8**	11.590

**Table 8:** Comparison of the generalization performance of MSP-Net on PASCAL VOC and MS COCO.

Model	Dataset	P/%	R/%	F1-Score	mAP@0.5/%	mAP@0.5:0.95/%	Params/M
YOLOv11s	COCO	67.4	54.3	60.1	59.4	43.3	**9.443**
**MSP-Net**	COCO	**68.9**	**54.4**	**60.7**	**60.1**	**44.1**	11.618
YOLOv11s	VOC	80.8	73.8	77.1	82.0	61.7	**9.420**
**MSP-Net**	VOC	**81.8**	**74.6**	**78.0**	**82.4**	**62.1**	11.594

**Table 9:** Performance comparison of models at different lightweight stages.

Model	P/%	R/%	F1-Score	mAP@0.5/%	mAP@0.5:0.95/%	Params/M
YOLOv11s	90.7	85.3	87.9	90.0	64.6	9.415
YOLOv11n	84.8	75.6	79.9	82.5	51.6	2.583
MSP-11n	87.5	79.0	83.0	85.6	55.4	3.134
**L-MSP-Net (Ours)**	88.6	80.1	84.1	86.1	55.9	2.313

**Table 10:** Progressive pruning results for MSP-11n.

Model	mAP@0.5/%	mAP@0.5:0.95/%	Params/M	GFLOPs
YOLOv11n	82.5	51.6	2.583	6.3
MSP-11n	85.6	55.4	3.134	8.2
10%	86.3	**56.8**	2.987	7.7
20%	86.3	56.3	2.838	7.2
30%	86.0	56.1	2.680	6.8
40%	**86.4**	56.2	2.579	6.4
50%	86.1	55.9	2.313	5.8
60%	85.4	54.6	2.275	5.6
70%	84.6	53.3	2.178	5.3
80%	84.0	52.5	2.003	5.0
90%	79.2	45.7	**1.912**	**4.7**

* The percentages in the table represent the pruning rate. For example, 10% indicates that the pruning rate is 10%.

## Data Availability

The raw data supporting the conclusions of this article will be made available by the authors on request.
